# QUALITY OF CARE IN A LONG-TERM CARE COMMUNITY

**DOI:** 10.1093/geroni/igz038.1871

**Published:** 2019-11-08

**Authors:** Laura A Murray, Melinda Heinz

**Affiliations:** Upper Iowa University, Fayette, Iowa, United States

## Abstract

Older adults may need to reside in long-term care facilities for additional assistance. However, research indicates differences in the quality of care. The purpose of this study was to conduct a naturalistic observation, recording factors affecting the quality of care residents received in a long-term care community. Over a three-week period, observations took place in the nursing home, assisted living, and memory care portions of the community. We predicted that there would be more issues negatively impacting quality of care in the nursing home area due to its medical model philosophy. Open-observations were recorded, coded, and analyzed for themes. Results indicated that the most significant issue influencing quality of care in all areas of the long-term care facility was communication (N = 57 recorded instances). Approximately 66% of recorded communication issues in the nursing home were negative compared to positive (25%) or neutral (8%) instances. Elderspeak was prevalent with staff using high pitched voices or saying “hun” to residents. At times, staff spoke too loudly to residents who did not have hearing impairment or would talk about residents in front of other residents, not taking into consideration privacy. In the memory care environment, positive examples were noted. Staff was friendly and worked together as a team, creating a positive work environment. Overall, results indicated staff members may need professional development in the area of communication, particularly staff working in the nursing home. In addition, reminding staff while it is their workplace it is also the resident’s home would be beneficial.

